# Using the Social-Local-Mobile App for Smoking Cessation in the SmokeFreeBrain Project: Protocol for a Randomized Controlled Trial

**DOI:** 10.2196/12464

**Published:** 2018-12-06

**Authors:** Francisco Jódar-Sánchez, Laura Carrasco Hernández, Francisco J Núñez-Benjumea, Marco Antonio Mesa González, Jesús Moreno Conde, Carlos Luis Parra Calderón, Luis Fernandez-Luque, Santiago Hors-Fraile, Anton Civit, Panagiotis Bamidis, Francisco Ortega-Ruiz

**Affiliations:** 1 Research and Innovation Group in Biomedical Informatics, Biomedical Engineering and Health Economy Institute of Biomedicine of Seville Virgen del Rocío University Hospital / Spanish National Research Council / University of Seville Seville Spain; 2 Smoking Cessation Unit Medical-Surgical Unit of Respiratory Diseases Virgen del Rocío University Hospital Sevilla Spain; 3 Centro de Investigación Biomédica en Red de Enfermedades Respiratorias Carlos III Institute of Health Madrid Spain; 4 Salumedia Tecnologías Sevilla Spain; 5 Department of Architecture and Computer Technology School of Computer Engineering University of Seville Sevilla Spain; 6 Department of Health Promotion School for Public Health and Primary Care Maastricht University Maastricht Netherlands; 7 Medical Physics Laboratory School of Medicine Aristotle University of Thessaloniki Thessaloniki Greece

**Keywords:** smoking cessation, mobile applications, randomized controlled trial, economic evaluation

## Abstract

**Background:**

Smoking is considered the main cause of preventable illness and early deaths worldwide. The treatment usually prescribed to people who wish to quit smoking is a multidisciplinary intervention, combining both psychological advice and pharmacological therapy, since the application of both strategies significantly increases the chance of success in a quit attempt.

**Objective:**

We present a study protocol of a 12-month randomized open-label parallel-group trial whose primary objective is to analyze the efficacy and efficiency of usual psychopharmacological therapy plus the Social-Local-Mobile app (intervention group) applied to the smoking cessation process compared with usual psychopharmacological therapy alone (control group).

**Methods:**

The target population consists of adult smokers (both male and female) attending the Smoking Cessation Unit at Virgen del Rocío University Hospital, Seville, Spain. Social-Local-Mobile is an innovative intervention based on mobile technologies and their capacity to trigger behavioral changes. The app is a complement to pharmacological therapies to quit smoking by providing personalized motivational messages, physical activity monitoring, lifestyle advice, and distractions (minigames) to help overcome cravings. Usual pharmacological therapy consists of bupropion (Zyntabac 150 mg) or varenicline (Champix 0.5 mg or 1 mg). The main outcomes will be (1) the smoking abstinence rate at 1 year measured by means of exhaled carbon monoxide and urinary cotinine tests, and (2) the result of the cost-effectiveness analysis, which will be expressed in terms of an incremental cost-effectiveness ratio. Secondary outcome measures will be (1) analysis of the safety of pharmacological therapy, (2) analysis of the health-related quality of life of patients, and (3) monitoring of healthy lifestyle and physical exercise habits.

**Results:**

Of 548 patients identified using the hospital’s electronic records system, we excluded 308 patients: 188 declined to participate and 120 did not meet the inclusion criteria. A total of 240 patients were enrolled: the control group (n=120) will receive usual psychopharmacological therapy, while the intervention group (n=120) will receive usual psychopharmacological therapy plus the So-Lo-Mo app. The project was approved for funding in June 2015. Enrollment started in October 2016 and was completed in October 2017. Data gathering was completed in November 2018, and data analysis is under way. The first results are expected to be submitted for publication in early 2019.

**Conclusions:**

Social networks and mobile technologies influence our daily lives and, therefore, may influence our smoking habits as well. As part of the SmokeFreeBrain H2020 European Commission project, this study aims at elucidating the potential role of these technologies when used as an extra aid to quit smoking.

**Trial Registration:**

ClinicalTrials.gov NCT03553173; https://clinicaltrials.gov/ct2/show/record/NCT03553173 (Archived by WebCite at http://www.webcitation.org/74DuHypOW).

**International Registered Report Identifier (IRRID):**

PRR1-10.2196/12464

## Introduction

### Background

Smoking is considered the main cause of preventable illness and early deaths worldwide [1]. Mathers and Loncar [2] estimated that about 100 million deaths were caused by tobacco addiction in the 20th century. Also, 5.4 million people worldwide die each year of tobacco-related diseases, and it is estimated that by 2030 smoking will cause 8 to 10 million deaths a year, over 80% of them in low- and middle-income countries [3]. According to the World Health Organization, in 2025, about 22% of the adult populations of Europe will be regular smokers [4]. Together with the Americas, Europe has the highest proportion of all deaths attributable to tobacco, estimated at 16% [1].

In Spain, in 2017, 22.08% of the population aged over 15 years smoked daily and 2.34% were occasional smokers. In Spain 25.58% of males and 18.76% of females are smokers; and 17.56% of young persons aged 15 to 24 years old have a tobacco habit, showing a relevant difference by sex (19.96% of males compared with 15.05% of females) [5].

In the Andalusian Health Service region, the largest public health problem is the smoking epidemic. To address this problem, a plan called “Comprehensive Tobacco Action Plan for Andalusia” has been defined and promoted [6] by the Andalusian Ministry of Health. This plan expects to reduce the incidence and prevalence of smoking in Andalusia, reducing complications and morbidity and mortality related to tobacco among the Andalusian population, and improving the quality of life of both smokers and nonsmokers. The plan hopes to create a smokefree future in a climate of social well-being and mutual respect, promoting healthy lifestyles, ensuring the right to health for all, and promoting public participation, with the final aim to guarantee smokers the best health care, based on scientific evidence, and ensuring continuity of care as an element of integral quality.

Smoking is usually considered to be exclusively a personal decision. This statement seems not to be true, since the clear majority of smokers claim that they wish to stop consuming tobacco when they are deeply aware of all the negative side effects to their health, yet they find it difficult to stop smoking due to the great addictiveness of nicotine. Fortunately, there are a variety of useful pharmaceutical products to help them quit. Among them, bupropion and varenicline are 2 drugs usually prescribed. At the Smoking Cessation Unit of the Virgen del Rocío University Hospital (VRUH) in Seville, Spain, patients willing to quit smoking are provided with a combination of both psychological advice and pharmacological treatment using any of the 2 previously mentioned drugs. This multidisciplinary strategy to quit has significantly improved the success rate [7].

Some research has been performed regarding the use of tailored mobile- and Web technology–based interventions and their impact on the smoking cessation process. Hébert et al [8] showed the utility of mobile phones for assessing the risk for smoking lapse in real time, and their findings endorsed the statement that tailored content may affect users’ urge to smoke, stress, and cigarette availability. Chakraborty et al [9] investigated the correlation between the personalization level of Web-based interventions and participants’ educational level when dealing with their smoking behavior, finding that highly individually tailored interventions were more effective for smokers with a low level of education. However, it is not clear what impact such interventions may have on smoking cessation efficacy in the long term. A recent observational study highlighted key insights related to participant engagement and cessation among adults who voluntarily subscribed to a 42-day mobile phone text message smoking cessation program [10], uncovering the need to improve program engagement. On the other hand, evidence showed a beneficial impact of mobile phone–based smoking cessation interventions on 6-month cessation outcomes, although caution should be taken in generalizing these results outside this type of intervention and context [11]. Nonetheless, there is still a need to demonstrate the added value that a tailored smoking cessation intervention based on mobile technologies has for the efficacy of long-term abstinence when added to psychopharmacological therapies.

Craving is a key component that has been shown to vary over time during a smoking cessation attempt and is highly related to treatment efficacy and cessation success [12]. In addition, it is documented that craving fades away during the first 2 weeks of abstinence [13]. However, cravings may return if former smokers’ coping strategies lose effectiveness over time, leaving smokers with less and less ability to resist the urge to smoke. There is scarce evidence about the valuable support that physical activity and information and communication technologies (app gamification, short text sent as push notifications, and short message service [SMS] text messaging and Facebook) may provide to the smoking cessation process [14,15].

### Objectives

As part of the SmokeFreeBrain H2020 European Commission project (Grant Agreement 681120) [16,17], this study aims at elucidating the potential role of the aforementioned technologies when used as an extra aid to quit. The Social-Local-Mobile (So-Lo-Mo) intervention focuses on providing health goals, including physical activity, with immediate feedback through the mobile app, reinforcing patients’ ability to stay abstinent with motivational messages and offering patients tools to overcome cravings, such as playing specifically designed minigames.

The main objectives of this study are to analyze the efficacy and cost effectiveness of usual psychopharmacological therapy plus the So-Lo-Mo app (intervention group) compared with usual psychopharmacological therapy alone (control group) applied to the smoking cessation process.

Secondary objectives are the following: (1) to analyze the safety of pharmacological therapy, (2) to analyze patients’ health-related quality of life (HRQoL), and (3) to monitor patients’ healthy lifestyle and physical exercise habits.

## Methods

### Design and Setting

This is a 12-month randomized open-label parallel-group trial performed at the VRUH. It was retrospectively registered on June 12, 2018 (NCT03553173).

### Participants and Recruitment Strategy

We calculated sample size during the study design phase according to the following parameters: (1) confidence level: 95%, (2) statistical power: 80%, (3) success rate in the control group: 35%, (4) success rate in the intervention group: 55%, and (5) expected dropout rate: 20%. This calculation yielded a sample size of N=236 participants. However, because this study was framed within a research project with tight deadlines for recruitment, we had only 12 months available for recruiting participants. Therefore, we calculated the sample size according to the average consultations performed by the Smoking Cessation Unit of VRUH during the last 5 years, which resulted in a slightly higher N=240.

Inclusion criteria are as follows: (1) the smoking population attending the Smoking Cessation Unit of VRUH, (2) age 18 years or older and the desire to give up smoking, (3) availability of an Android-based mobile phone, (4) ability to interact with the mobile phone (we will assess mobile phone literacy by asking patients if they commonly use other text exchange mobile phone apps such as Mail, SMS, or WhatsApp), and (5) willingness to sign an informed consent form.

We excluded patients who had some previous adverse effects related to the pharmacological treatment used in the study.

It should be noted that we will use the allocation to different pharmacological therapies as a reflection of usual care rather than a confounding factor for the analysis. We generated a list of 240 consecutive items by randomly assigning 1 of the study groups to each item. Participants were assigned to each of the study groups according to this list and following their order of enrollment.

We obtained written informed consent from all study participants.

### Usual Psychopharmacological Therapy

Usual care consists of pharmacological therapy with bupropion (Zyntabac 150 mg; Glaxosmithkline SA, Tres Cantos, Spain) or varenicline (Champix 0.5 mg or 1 mg; Pfizer SL, San Sebastián de los Reyes, Spain) and behavioral therapy. In routine care, patients must pay for these treatments in the pharmacy. To facilitate patient recruitment and to avoid bias due to the treatment cost, the SmokeFreeBrain project is funding these costs, so participants receive the assigned treatment free of charge.

The psychological intervention process, which is performed individually, starts with an assessment of the smoker to complement his or her medical record. The first step is to assess the patient’s smoking record (number of daily cigarettes, age at the start of regular smoking, previous quit attempts, and methods followed for quitting). The next step is to assess the patient’s nicotine dependence level using the Fagerström Test for Nicotine Dependence [18] and motivation to quit smoking according to the Richmond test [19].

The psychological intervention starts once these assessments have been performed. This intervention includes providing information about smoking and the smoking cessation process, as well as supporting behavioral changes by providing new skills and strategies. Although there is a wide variety of psychological interventions to support the smoking cessation process, such as group behavior therapy programs and self-help materials [20], the most used methods delivered by the Smoking Cessation Unit of the VRUH are the motivational interview and cognitive behavioral therapy.

The motivational interview is performed in 2 stages—exploratory and decisive—and includes the following basic principles: (1) to express empathy, (2) to create discrepancy, (3) to overcome resistance, (4) to avoid arguing, and (5) to encourage self-efficacy.

The objective of cognitive behavioral therapy is to reconvert the smoker’s thoughts, knowledge, and behavior related to smoking. To achieve this, the following are applied: (1) problem solving skills training, (2) social support (by phone and email), (3) self-analysis of reasons to quit, (4) cigarette consumption registry, (5) progressive reduction of cigarette consumption, and (6) relapse prevention strategies.

### The Social-Local-Mobile Intervention

So-Lo-Mo is an innovative intervention based on mobile technologies and their capacity to trigger behavioral changes. In this sense, the app is a complement to pharmacological therapies to quit smoking providing, among other features, personalized motivational messages sent by the system, peer-to-peer messaging, physical activity monitoring, lifestyle advice, and distractions (minigames) to help overcome cravings. The main objective of this app is to improve patients’ adherence to the smoking cessation process by use of behavioral techniques in the form of motivational messages and SMS text messages. To this aim, the underlying algorithm is able to do the following:

- Send motivational messages as SMS text messages or in a notification-based format. High-priority messages—those related to specific dates such as New Year’s Eve or holidays when people may relapse—are sent by SMS so that their delivery is guaranteed even when users do not have an internet connection on their mobile, while the rest of the messages are delivered via app push notification mechanisms, which uses an internet connection.
- Dynamically schedule message frequency according to the phase of the transtheoretical model of behavior change [21] that the individual is undergoing (preparation, action, or maintenance) and when the notifications should be delivered according to the individual’s preferences.
- Dynamically determine the category (ie, content and type) of the message that is delivered to the individual according to the phase of the transtheoretical model of behavior change that the individual is undergoing, user health conditions, user feedback, and user filtering strategies. For this, we have defined the following motivational message categories: reduce tobacco consumption, increase risk perception, and increase benefit perception during the preparation phase; and general motivation, diet tips, exercise and active life recommendations, personal physical activity level, and positive facts of being a former smoker during the action and maintenance phases.

**Figure 1:**
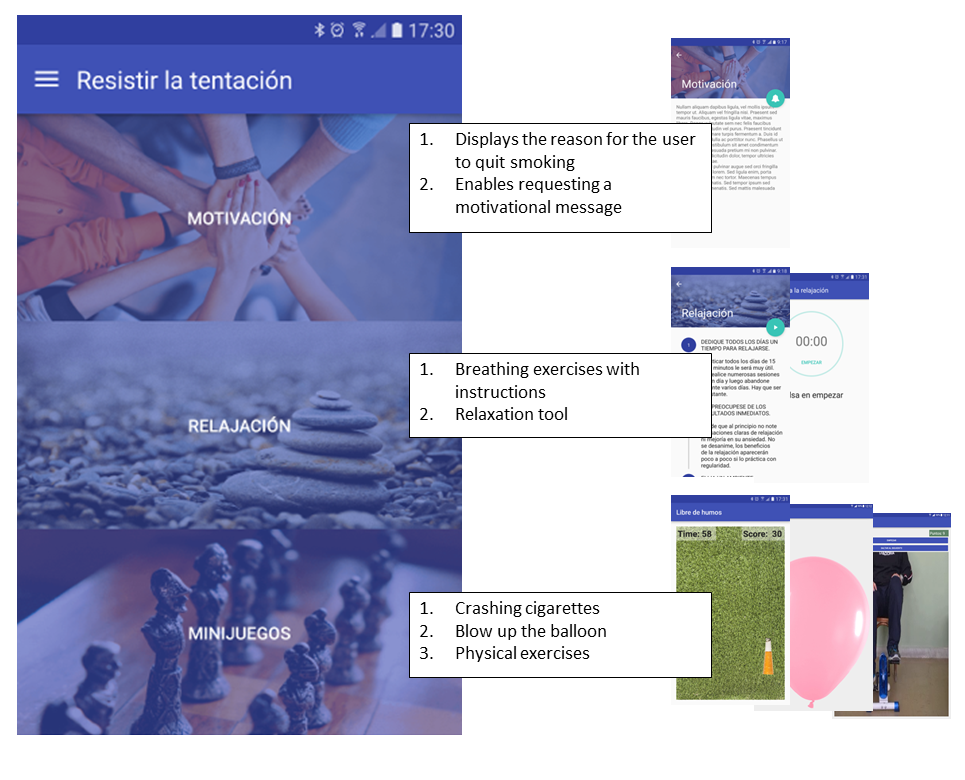
Sections of the app that address how to resist cravings.

Figure 1 shows the 3 specific sections of the app that address how to resist cravings: (1) motivational messages that the user can request immediately from the system, (2) relaxation tools such as breathing exercises, and (3) minigames specifically designed to help the user overcome cravings. Hors-Fraile et al [22] provide further information on the methods and the underlying algorithm for delivering these text messages.

### Study Outcomes

The main outcomes will be the efficacy and efficiency of the So-Lo-Mo intervention.

To assess efficacy, the main clinical outcome will be the smoking abstinence rate at 1 year measured by means of exhaled carbon monoxide and urine cotinine tests.

Exhaled carbon monoxide is part of the smoke and can be measured by a carbon monoxide tester. The patient must perform a deep inspiration and hold the air for 15 seconds. Then, the patient must exhale the air inside the carbon monoxide tester (Micro+ Smokerlyzer; Bedfont Scientific Ltd, Maidstone, UK) in a slow, sustained, and complete fashion. The carbon monoxide tester then yields the exact amount of exhaled carbon monoxide in parts per million. Exhaled carbon monoxide levels are highest between 3 and 6 hours after smoking a cigarette. A person is considered to be smoker when his or her exhaled carbon monoxide is higher than 6 ppm [23].

The urine cotinine (SmokeScreen test; GFC Diagnostics Ltd, Chipping Warden, UK) is a colorimetric test that measures the main metabolites of nicotine, including cotinine. In Spain, patients with cotinine concentrations over 200 ng/mL are considered to be smokers [24].

To consider a participant to be a smoker, only 1 of the aforementioned conditions needs to be met.

To assess efficiency, we will carry out a cost-effectiveness analysis considering the recommendations of the proposed guidelines for economic evaluation of health technologies [25]. The analysis will adopt the perspective of the Spanish National Health System. We will express results of the cost-effectiveness analysis in terms of the incremental cost-effectiveness ratio, calculated by dividing the difference in total costs between the intervention group and the control group by the difference in quality-adjusted life-years (QALYs) between the 2 groups [26]. Cost analysis will include costs of the prescribed medication, time spent by the health professionals on the So-Lo-Mo intervention, health care resources utilization, and equipment and software costs related to the So-Lo-Mo intervention. We will calculate QALYs in order to assess the health benefit of the intervention regarding the cost-effectiveness analysis. QALY is a health outcome that includes both the quality and the quantity of life, and we will calculate it through 5-level EuroQol 5 dimensions questionnaire (EQ-5D-5L) scores [27], according to the Spanish validation [28].

The secondary outcomes will be safety, patients’ HRQoL, and patients’ healthy lifestyle and exercise habits.

We will measure safety as the number of adverse events related to pharmacological therapies. The following adverse events have been identified related to each pharmacological therapy: (1) for varenicline (Champix 0.5 mg or 1 mg): nausea (feeling sick), insomnia (difficulty sleeping), abnormal dreams, headache, and nasopharyngitis (inflammation of the nose and throat) [29]; and (2) for bupropion (Zyntabac 150 mg): insomnia, headache, dryness in the mouth (alteration of taste), skin reactions, convulsions, cardiovascular side effects, and severe skin reactions [30].

We will measure HRQoL through the EQ-5D-5L questionnaire [27] and the 36-item Short Form Health Survey [31].

We will monitor physical activity through the International Physical Activity Questionnaire [32] and healthy lifestyle in terms of the variation of body mass index during the follow-up consultations.

Moreover, information from all patients undergoing this study will include demographic data (age and sex) and socioeconomic data (profession and employment status); consumption history (eg, daily cigarettes, living with smokers, partner smokers, quit attempts); clinical information (eg, weight, size, blood pressure, comorbidities); and nicotine dependence measured through the Fagerström Test for Nicotine Dependence [18]. Multimedia Appendix 1 shows every variable of the evaluation framework and its use in each follow-up session.

### Data Collection

**Figure 2:**
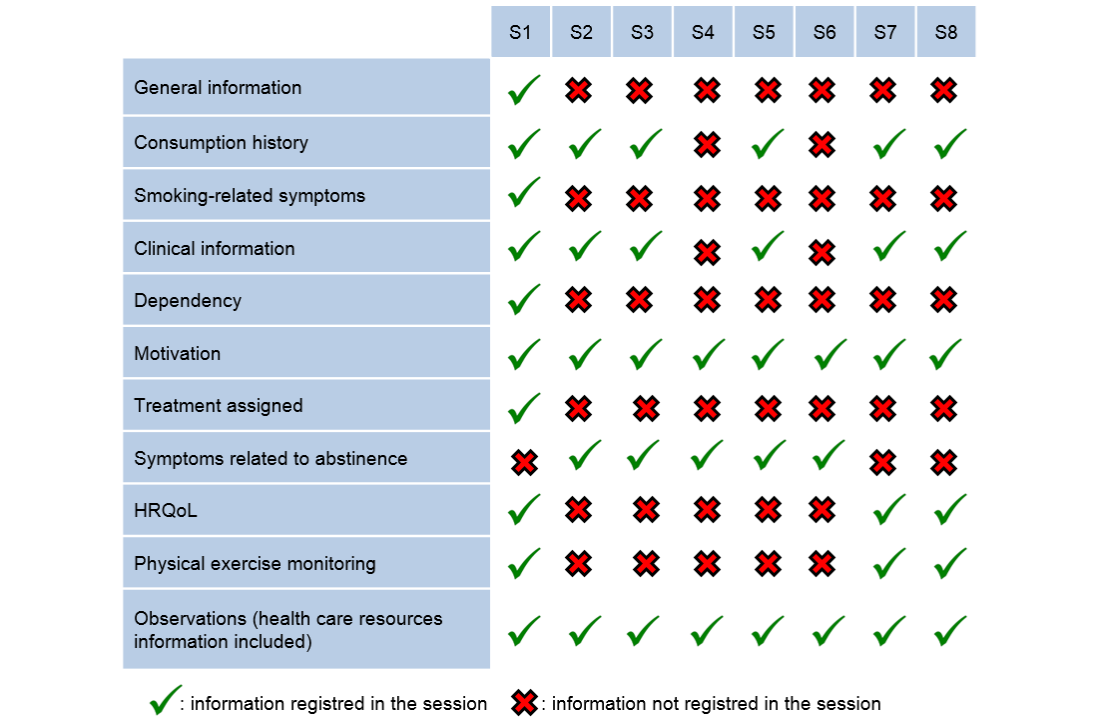
Information subsets and follow-up schedule to record in the study clinical database. HRQoL: health-related quality of life; S1-S8: sessions 1-8.

We will record information subsets in the So-Lo-Mo clinical database according to the following schedule (Figure 2):

In session 1 (baseline), patients are assessed for the first time in the Smoking Cessation Unit as they are referred from either the Pneumology Unit or another clinical department. We will collect information regarding the following sections: general information, consumption history, smoking-related symptoms, clinical information, dependency, motivation, treatment assigned, HRQoL, physical exercise monitoring, and observations.

In session 2, 15 (±5) days after the baseline consultation, we will collect information regarding the following sections: consumption history, clinical information, motivation, symptoms related to abstinence, and observations. Relaxation techniques and risk-avoidance techniques will also be explained to the patient.

In session 3, 30 (±5) days after the baseline consultation, we will collect information regarding the following sections: consumption history, clinical information, motivation, symptoms related to abstinence, and observations. New relaxation techniques will be explained in case those previously explained proved to be useless. Patients will be coached for relapse prevention.

In session 4, 60 (±5) days after the baseline consultation, we will collect information regarding the following sections: motivation, symptoms related to abstinence, and observations. Risk-avoidance techniques will be reinforced.

In session 5, 90 (±5) days after the baseline consultation, we will collect information regarding the following sections: consumption history, clinical information, motivation, symptoms related to abstinence, and observations. Patients will be coached for relapse prevention and confrontational techniques will be explained.

In session 6, 120 (±5) days after the baseline consultation, patients can be assessed by phone if they have completed the pharmacological treatment. In this session we will collect information regarding motivation, symptoms related to abstinence, and observations. Patients will be coached for relapse prevention and confrontational techniques will be explained.

In session 7, 180 (±5) days after the baseline consultation, we will collect information regarding the following sections: consumption history, clinical information, motivation, HRQoL, physical exercise monitoring, and observations (including information on health care resources). Relaxation and confrontational techniques will be reinforced when needed.

In session 8, 365 (±5) days after the baseline consultation, we will collect information regarding the following sections: consumption history, clinical information, motivation, HRQoL, physical exercise monitoring, and observations (including information on health care resources).

We will develop a case report form built on the OpenClinica [33] tool to facilitate information management in the study. It is worth noting that OpenClinica is compliant with the Guideline for Good Clinical Practice, US Code of Federal Regulations, Title 21, Part 11, the US Health Insurance Portability and Accountability Act of 1996, and other regulations. This case report form is integrated with the VRUH’s electronic health record system, so some data elements are automatically loaded into the case report form, thus avoiding double recording for clinicians.

### Statistical Analysis

At the end of the study, we will carry out a descriptive analysis of patients’ characteristics by absolute and relative frequencies for qualitative variables and mean (SD) for quantitative variables. We will perform a bivariate analysis of study groups for qualitative variables by chi-square test or Fisher exact test. Comparison of quantitative variables will differ depending on technical assumptions, such as parametric (Student *t* test or analysis of variance) or nonparametric (Mann-Whitney *U* or Kruskal-Wallis) tests.

To analyze the uncertainty of the incremental cost-effectiveness ratio results, we will provide a tornado diagram of the univariate sensitivity analysis, incorporating variations in the components of cost and QALY. The data analysis team will not be blinded to the allocation of participants.

## Results

**Figure 3:**
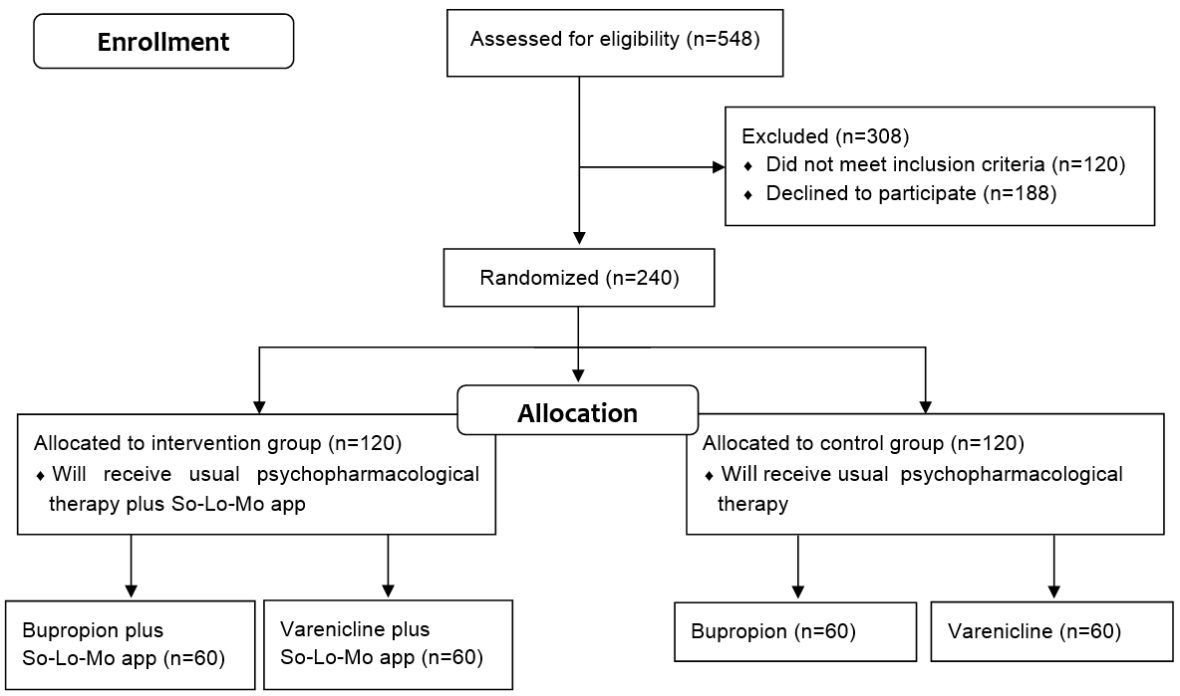
Flow diagram of the randomized open-label parallel-group trial of the Social-Local-Mobile (So-Lo-Mo) app.

We identified 548 patients using the hospital’s electronic records system. From this initial selection, we excluded 308 patients: 188 declined to participate (149 did not show up for the baseline consultation, 27 refused the medication, 7 did not want to enroll in the study, and 5 did not want to quit smoking), while 120 did not meet the inclusion criteria (98 reported previous adverse effects related to the medication assigned, 15 were not smokers at baseline, and 7 did not have an Android-based smartphone available). A total of 240 patients were enrolled: the control group (n=120) will receive usual psychopharmacological therapy, while the intervention group (n=120) will receive usual psychopharmacological therapy plus the So-Lo-Mo app. Figure 3 shows the enrollment and allocation phases of the study.

The project was approved for funding in June 2015. Enrollment started in October 2016 and was completed in October 2017. Data gathering was completed in November 2018 and data analysis is under way. We expect to submit the first results for publication in early 2019.

Virgen Macarena–Virgen del Rocío University Hospitals Ethics Committee approved the study protocol in July 2016.

## Discussion

Nowadays, the treatment usually prescribed to patients who wish to give up smoking is a multidisciplinary intervention, combining both psychological advice and pharmacological therapy, since the application of both strategies significantly increases the chance of success in a quit attempt [34]. Psychological interventions can be performed at different levels depending on resource availability and the level of care, without a difference in efficacy between individual and group therapies. In this scenario, psychological treatments are based on confrontational techniques, including behavioral and cognitive behavioral therapies [35]. Behavioral therapies are designed to help smokers to recognize and avoid external stimuli temporarily associated with the consumption of tobacco, while cognitive behavioral therapies provide the tools to confront both physiological and cognitive stimuli with the urge to smoke. During the smoking cessation process, 1 in 3 smokers rely on the pharmacological treatment too [36]. There is a wide range of drugs that could support this process, and the physicians in the Smoking Cessation Unit at VRUH usually prescribe either varenicline or bupropion. Varenicline, bupropion, and nicotine replacement therapy reduce-to-quit interventions have all been found to be effective cessation interventions in smokers who would like to quit [37].

A recent systematic review of smartphone apps for smoking cessation highlighted that future studies should aim to develop and standardize an innovative and timely approach to evaluate apps for commitment to evidenced-based practice; to explore strategies to make scientifically supported apps easily searchable and more accessible to consumers, including indexing with plain language terminology; and to explore ways to inform the development of health apps to better align app content with evidence-based medicine [38].

In this sense, we will compare the efficacy of usual pharmacological treatment plus behavioral and cognitive behavioral therapies routinely delivered at VRUH when added to the So-Lo-Mo intervention. If adding the So-Lo-Mo intervention provides any meaningful increase in the smoking cessation treatment effectiveness rate, we can infer that this increment will also take place when added to nicotine replacement therapy, given that it is not as effective as varenicline or bupropion. Furthermore, we are conducting a parallel study to assess the perceived quality of the health recommender system and the level of engagement with the motivational messages. The analysis of these technical aspects will provide a more comprehensive understanding of the So-Lo-Mo intervention [39].

Cultural and material factors are also key elements that need to be further investigated to uncover their correlation with smoking habit in different geographical regions [40,41]. Recently, the Taipei Medical University joined the SmokeFreeBrain project and they are currently developing an intervention similar to So-Lo-Mo [42]. Their involvement reflects not only the capacity of this intervention to cross borders and go beyond the frame of a European project, but will also shed some light on the cultural grounding of the efficacy and effectiveness of the use of mobile technologies applied to the smoking cessation process.

Understanding the way social networks and mobile technologies can influence individual behavior and the decision of whether to smoke is essential to optimize their use as a means of prevention and treatment in the future. A recent scoping review [43] showed how health recommender systems, like the one used in So-Lo-Mo, are being used in health care with many potential benefits for their users. However, most of the systems lacked any description of their design, follow-up of implementation, and behavioral change models they were based on. So-Lo-Mo is, thus, an innovative intervention based on mobile technologies and its capacity to trigger behavioral changes following the transtheoretical behavioral change model [21].

Although a large number of studies have assessed the effects of interventions intended to reduce the harm to health of continued tobacco use, it is important that more high-quality randomized controlled trials be conducted, and that these also measure the long-term health effects of treatments [44]. Another innovative contribution of our study is the focus on the cost-effectiveness analysis that will consider the economic impact on the Spanish National Health System and on patients’ HRQoL.

Some limitations of our study should be acknowledged. First, the So-Lo-Mo app is developed for Android-based users only. However, in Spain the market share for Android-based devices in 2018 is 78.09% [45]. This means that only a small percentage of patients will be excluded from the study due to this criterion. Second, we did not calculate the sample size based on power calculations, as explained above, which will limit our ability to extract strong evidence about the efficacy and efficiency of the intervention applied to the smoking cessation process after completion of the study.

## Appendix

Multimedia Appendix 1 Variables of the evaluation framework.

Multimedia Appendix 2 Peer-reviewer report from the European Commission.

## Abbreviations

EQ-5D-5L: 5-level EuroQol 5 dimensions questionnaire
HRQoL: health-related quality of life
QALY: quality-adjusted life-year
SMS: short message service
So-Lo-Mo: Social-Local-Mobile
VRUH: Virgen del Rocío University Hospital
