# Supplementary material for: Using the Social-Local-Mobile App for Smoking Cessation in the SmokeFreeBrain Project: Protocol for a Randomized Controlled Trial
Source: JMIR Res Protoc. 2018 Dec 6;7(12):e12464. doi: 10.2196/12464 (PMC6302230; doi:10.2196/12464)
Supplement: Multimedia Appendix 2 [file resprot_v7i12e12464_app2.pdf]

Proposal Evaluation Form

EUROPEAN COMMISSION<br>Horizon 2020 - Research and Innovation Framework Programme	Evaluation Summary Report - Research and innovation actions/Innovation actions

**Call:**	H2020-HCO-2015
**Funding scheme:**	Research and Innovation action
**Proposal number:**	681120
**Proposal acronym:**	SmokeFreeBrain
**Duration (months):**	36
**Proposal title:**	Multidisciplinary tools for improving the efficacy of public prevention measures against smoking
**Activity:**	HCO-06-2015

N.	Proposer name	Country	Total Cost	%	Grant Requested	%
1	ARISTOTELIO PANEPISTIMIO THESSALONIKIS	EL	633,750	21.26%	633,750	21.26%
2	UNIVERSITY OF SURREY	UK	438,500	14.71%	438,500	14.71%
3	Department of Health	UK	246,500	8.27%	246,500	8.27%
4	AAI SCIENTIFIC CULTURAL SERVICES LIMITED	CY	340,626	11.43%	340,626	11.43%
5	UNIVERSITA DEGLI STUDI DI ROMA LA SAPIENZA	IT	317,050	10.64%	317,050	10.64%
6	SERVICIO ANDALUZ DE SALUD	ES	242,500	8.13%	242,500	8.13%
7	National association of General practitioners in Bulgaria	BG	121,250	4.07%	121,250	4.07%
8	Klados Manousos & SIA E.E.	EL	216,250	7.25%	216,250	7.25%
9	HELLENIC OPEN UNIVERSITY	EL	88,125	2.96%	88,125	2.96%
10	Salumedia Tecnologías S.L.	ES	80,625	2.70%	80,625	2.70%
11	INSTITUT ZA PLUCNE BOLESTI VOJVODINE	RS	120,200	4.03%	120,200	4.03%
12	UNIVERSIDAD DE SEVILLA	ES	135,625	4.55%	135,625	4.55%
	Total:		2,981,001		2,981,001	

Abstract:
Smoking is the largest avoidable cause of preventable morbidity worldwide. It causes most of the cases of lung cancer and chronic obstructive pulmonary disease (COPD) and contributes to the development of other lung diseases. The control of smoking is considered as a highly important intervention for the prevention of lung diseases. Tobacco consumption is highly influenced by socioeconomic factors. SmokeFreeBrain aims to address the effectiveness of a multi-level variety of interventions aiming at smoking cessation in high risk target groups within High Middle Income Countries (HMIC) such as unemployed young adults, COPD and asthma patients, as well as within the general population in Low Middle Income Countries (LMIC). The project addresses existing approaches aiming to prevent lung diseases caused by tobacco while at the same time it develops new treatments and analyzes their contextual adaptability to the local and global health care system. SmokeFreeBrain follows an interdisciplinary approach exploiting consortium's expertise in various relevant fields in order to generate new knowledge. State of the art techniques in toxicology, pulmonary medicine, neuroscience and behavior will be utilized to evaluate the effectiveness of: (i) Public Service Announcement (PSA) against smoking, (ii) the use of electronic cigarettes with and without nicotine as a harm reduction approach and/or cessation aid, (iii) a specifically developed neurofeedback intervention protocol against smoking addiction, (iv) a specifically developed intervention protocol based on behavioral therapy, social media/mobile apps and short text messages (sms) and (v) pharmacologic interventions. The main objective of the project is to evaluate the interventions in terms of health economics, by studying their cost-effectiveness, and proposing a scalable plan and a clear pathway to embedding the proposed interventions into policy and practice both in LMIC as well as in HMIC.

Evaluation Summary Report

Evaluation Result

Total score: 13.00 (Threshold: 10)

Form information

SCORING

Scores must be in the range 0-5.

Interpretation of the score:

*0 The **proposal fails to address the criterion** or cannot be assessed due to missing or incomplete information.*

***1 Poor.** The criterion is inadequately addressed, or there are serious inherent weaknesses.*

***2 Fair.** The proposal broadly addresses the criterion, but there are significant weaknesses.*

***3 Good.** The proposal addresses the criterion well, but a number of shortcomings are present.*

***4 Very good.** The proposal addresses the criterion very well, but a small number of shortcomings are present.*

***5 Excellent.** The proposal successfully addresses all relevant aspects of the criterion.Any shortcomings are minor.*

Criterion 1 - Excellence

Score: **4.00** (Threshold: 3/5.00 , Weight: 100.00%)

Note: The following aspects will be taken into account, to the extent that the proposed work corresponds to the topic description in the work programme. If a proposal is partly out of scope, this must be reflected in the scoring, and explained in the comments.
Clarity and pertinence of the objectives
Credibility of the proposed approach
Soundness of the concept, including trans-disciplinary considerations, where relevant
Extent that proposed work is ambitious, has innovation potential, and is beyond the state of the art (e.g. ground-breaking objectives, novel concepts and approaches)

Strengths:

• The proposal fits the work programme. The proposal is clear enough and it is described as a multilevel intervention (it involves pulmonary diseases, toxicology, neuroscience, EEG, behavioural measurements, Public Service Announcements) aimed at high risk populations in HIC and general population in LMIC.
• Soundness of the concept is relevant. The proposal addresses as well a neuroscientific approach to smoking prevention and cessation. At the same time, the toxicology study about e-cigarettes in animals and in humans is very good.
• The objectives are clearly described and the different sub projects are well explained.
• The proposal is ambitious, has innovation potential and is beyond the state of the art.
• The proposed approaches are credible.

Weaknesses:

• Neurofeedback section: In the reviewers' opinion, the section related to brain function regulated in a natural way is lacking key arguments about the planned way to deal with several obstacles making this procedure unsuitable as a part of the targeted prevention policy for lung diseases.
• 1.1.2.3. Social Media: So-Lo-Mo (Social-Local-Mobile): this tool that applicants would like to use involves some risks in a long term due to high frequency of substance and behavioural addiction comorbidity. It should be emphasized that a strong relationship exists between the use of tobacco and high risk of Internet addiction (including Internet Gaming Disorder). In fact, recent research shows that lifetime use of tobacco predicted high risk of Internet addiction (see for instance: Evren C et al. Psychiatr Danub. 2014 Dec;26(4):330-9.) Additionally, the two disorders share the same neural substrates (Ko et al. J Psychiatr Res. 2013 Apr;47(4):486-93). Thus, there is a specific risk in using an online app based on gamification for people vulnerable to develop an Internet addiction, such as smokers.

Criterion 2 - Impact

Score: **4.00** (Threshold: 3/5.00 , Weight: 100.00%)

Note: The following aspects will be taken into account, to the extent to which the outputs of the project should contribute at the European and/or International level:
The expected impacts listed in the work programme under the relevant topic
Enhancing innovation capacity and integration of new knowledge
Strengthening the competitiveness and growth of companies by developing innovations meeting the needs of European and global markets, and where relevant, by delivering such innovations to the markets
Any other environmental and socially important impacts
Effectiveness of the proposed measures to exploit and disseminate the project results (including management of IPR), to communicate the project, and to manage research data where relevant

Strengths:

• The development of effective smoking interventions based on cost effectiveness can have a high practical impact.
• The proposal will generate important new knowledge on specific interventions (e.g. neurofeedback, PSA, e-cigarette). Moreover, the objectives related to e-cigarette, Public Service Announcements and Pharmacology could fill some gaps in the relative field.
• The strategies for dissemination and exploitation of results and the communication activities are excellent. The results of the project will be presented to policymakers, the scientific community, clinicians, the general population, patient groups and the industry. The concept for communicating the project and its results is well-balanced and thought out. Excellent is that the coordinator has already contacted national and international organizations to maximize the impact of the project.
• Performance indicators for dissemination measures are well described.

Weaknesses:

• There are some concerns whether an intervention via mobile app will contribute to the specific expected impact set out in the work programme: "To reduce health inequalities and inequities, including gender, in the prevention and treatment of lung diseases in both a local and global context", especially for LMIC countries where the use of the Internet is still low. The reviewers expressed concerns regarding people who do not have access to the Internet such as people unemployed or living in poverty (both risk factors for smoking and lung diseases) that may not benefit from the hypothetical advantages of such intervention.
• The study on Neurofeedback does not plan to provide data on abstinence.

Criterion 3 - Quality and efficiency of the implementation

Score: **5.00** (Threshold: 3/5.00 , Weight: 100.00%)

Note: The following aspects will be taken into account:
Coherence and effectiveness of the work plan, including appropriateness of the allocation of tasks and resources
Complementarity of the participants within the consortium (when relevant)
Appropriateness of the management structures and procedures, including risk and innovation management

Strengths:
• The projects partners will work together and will complement each other.
• The management procedures are appropriate as well as the allocation of tasks and resources.
• The work packages are well described and the evaluation of the obtained results is properly planned.
• The risk management is well-balanced.

The work packages are well described, especially for neurofeedback and e-cigarettes. The evaluation of the obtained results is properly planned. At the same time, the description of the pilot trials is very good. The methodology and tools are appropriate to measure the outcomes. In conclusion the quality and efficiency of the implementation of this proposal is excellent.

Weaknesses:
• Only minor weaknesses related to the distribution of the tasks, were identified.

Operational Capacity

Status: **Operational Capacity: Yes**

Not provided

Proposal content corresponds, wholly or in part, to the topic description against which it is submitted, in the relevant work programme part

Status: **Yes**

Not provided
